# Neuronal and non-neuronal signals regulate *Caernorhabditis elegans* avoidance of contaminated food

**DOI:** 10.1098/rstb.2017.0255

**Published:** 2018-06-04

**Authors:** Alexandra Anderson, Rachel McMullan

**Affiliations:** 1 Department of Life Sciences, Imperial College London, London SW7 2AZ, UK; 2 School of Life, Health and Chemical Sciences, The Open University, Milton Keynes, Buckinghamshire MK7 2AA, UK

**Keywords:** *Caenorhabditis elegans*, avoidance behaviour, host–pathogen interaction

## Abstract

One way in which animals minimize the risk of infection is to reduce their contact with contaminated food. Here, we establish a model of pathogen-contaminated food avoidance using the nematode worm *Caernorhabditis elegans*. We find that avoidance of pathogen-contaminated food protects *C. elegans* from the deleterious effects of infection and, using genetic approaches, demonstrate that multiple sensory neurons are required for this avoidance behaviour. In addition, our results reveal that the avoidance of contaminated food requires bacterial adherence to non-neuronal cells in the tail of *C. elegans* that are also required for the cellular immune response. Previous studies in *C. elegans* have contributed significantly to our understanding of molecular and cellular basis of host–pathogen interactions and our model provides a unique opportunity to gain basic insights into how animals avoid contaminated food.

This article is part of the Theo Murphy meeting issue ‘Evolution of pathogen and parasite avoidance behaviours’.

## 1. Introduction

Almost all animals are at risk from infection by pathogens and parasites in their environment. Consequently, animals have evolved tolerance, resistance and avoidance mechanisms to manage this constant threat. While we know a great deal about the immunology, biochemistry and genetics of infections, we know little about the pathogen avoidance behaviours, which prevent or reduce contact with pathogens.

As highlighted in this issue, pathogen and parasite avoidance behaviours have been observed experimentally in a diverse range of species including worms, ants, flies, bees, marine crustaceans, birds, marsupials, rodents and non-human primates. Of these, invertebrate model organisms such as the fruit fly *Drosophila melanogaster* and the nematode worm *Caenorhabditis elegans* provide a unique opportunity to use genetics in order to identify the evolutionarily conserved genes involved in pathogen avoidance. Furthermore, the ability to experimentally manipulate pathogen avoidance in these models allows us to examine the impact of this behaviour on infection.

The nematode worm *C. elegans* can be easily infected with a wide range of bacterial pathogens by providing them as a food source [1,2] and has evolved behavioural and cellular defences against a number of pathogens [3,4], providing a convenient model for the genetic dissection of pathogen avoidance. With only 302 neurons and 7000 synapses, the simple, well-described nervous system of *C. elegans* is frequently used to study complex behaviours at the level of genes, neurons and neural circuits [5]. Despite its simplicity, almost all gene families involved in mammalian neuronal function are conserved and behavioural changes, evoked by cues in the environment, can be easily quantified.

*C. elegans* uses multiple behavioural processes including innate and learned olfactory preference [6,7], mechanosensation [8] and aerotaxis [9] to avoid pathogenic strains of several bacteria, including *Serratia marcescens* [10,11], *Bacillus thuringiensis* [12], *Streptomyces* spp. [13], *Pseudomonas aeruginosa* [9] and *Microbacterium nematophilum* [14,15]. In the case of *P. aeruginosa*, this pathogen avoidance behaviour has been demonstrated to protect *C. elegans* from infection because mutant *C. elegans* that are unable to avoid pathogenic lawns are more susceptible to infections [9,16] while manipulating experimental conditions such that wild-type animals are unable to avoid pathogenic lawns also results in increased infection susceptibility [16].

Using genetic and biochemical approaches, some of the microbial cues, neurons and signalling pathways that mediate the avoidance of these pathogens have been identified [4]. G-protein signalling in the chemosensory neuron AWB [11] and signalling via the single *C. elegans* Toll-like receptor *tol-1* [10] are required to mediate the avoidance of pathogenic *S. marcesecens* with AWB being required to mediate an innate aversion to the bacterial surfactant Serrawettin W2 [11]. In the case of *B. thuringiensis*, the toxin Cry6A2a promotes the avoidance of bacterial lawns [17], and both neuropeptide [18] and insulin-like signalling [12] have been implicated in the regulation of this avoidance behaviour although the neuronal basis of this response remains to be identified. By contrast, several chemosensory neurons have been identified as required for the avoidance of *P. aeruginosa* and *Streptomyces* spp. [6,13]. In the case of *Streptomyces*, the G-protein-coupled receptor *srb-6* is required in chemosensory neurons ASH, ADL, ADF or AWA to detect dodecanoic acid produced by *Streptomyces* and promote avoidance [13]. A neuronal circuit involving AWC and AWB mediates the innate olfactory preference for *P. aeruginosa* resulting in initial attraction to *P. aeruginosa* lawns; however, in animals trained with pathogenic *P. aeruginosa*, serotonin signalling in ADF sensory neurons alters the downstream output of this circuit to promote pathogen avoidance [6]. Additionally, changes in aerotaxis and mechanosensation have been associated with pathogen avoidance behaviour [8,9]. Bacterial secondary metabolites activate G-protein signalling in the sensory neuron ASJ to mediate neuroendocrine signalling to alter aerotaxis behaviour and promote pathogen avoidance [19] while ablation of the mechanosensory neuron OLL results in enhanced avoidance of *P. aeruginosa*, suggesting that mechanosensory detection of bacteria may contribute to pathogen avoidance [8]. Lastly, *C. elegans* avoids the nematode-specific pathogen *M. nematophilum* [14,15]. The cyclic nucleotide-gated channel encoded by *tax-2* and *tax-4* is required for the avoidance of *M. nematophilum* because *tax-2(p671)* and *tax-4(e2861)* mutants are defective in this avoidance response and are unable to distinguish between *Escherichia coli* and *M. nematophilum* in a choice assay [14]. The *tax-2* and *tax-4* channel is essential for the function of many sensory neurons implicating chemosensation in this avoidance response, although the identity of the neurons required for this avoidance response remains unknown.

These previous studies have largely used either *C. elegans* propagated monoaxenically on pathogenic bacteria or bacterial choice assays in which animals chose between monoaxenic lawns of pathogenic and non-pathogenic bacteria. Here, we extend our understanding of the molecular and cellular basis of pathogen avoidance by investigating the response of *C. elegans* to food contaminated with pathogenic bacteria. Using lawns of *C. elegans* laboratory food source, *E. coli* OP50 contaminated with *M. nematophilum*, we show that *C. elegans* is also able to detect and avoid low levels of pathogenic bacteria in the presence of attractive cues from food. This avoidance behaviour decreases pathogen load and protects animals from the growth delay caused by this pathogen contamination. We further show that avoidance of contaminated bacterial lawns requires signalling in multiple sensory neurons as well as pathogen attachment to the non-neuronal cells associated with the cellular immune response to *M. nematophilum*. Our results establish an additional model of pathogen avoidance in *C. elegans*, which allows the avoidance of pathogen-contaminated food to be studied under controlled experimental conditions*.* Further investigation of this model will reveal additional insights into the genetics of pathogen avoidance and its role in protecting host species from infection.

## 2. Material and methods

### (a) Strains

*C. elegans* strains used in this study are detailed in electronic supplementary material, table S1. All strains were cultivated at 20°C on nematode-growth media (NGM) plates seeded with *E. coli* OP50, unless otherwise stated, and maintained as described previously [20].

### (b) *M. nematophilum* infection

Infection with *M. nematophilum* was performed as described previously [21]. NGM plates were seeded with 200 µl of 10% *M. nematophilum* (CBX102) or non-pathogenic *M. nematophilum* (UV336) diluted in *E. coli* OP50. Where indicated, CBX102 and UV336 were heat-inactivated by incubating at 65°C for 30 min. To test the effectiveness of heat-inactivation bacteria were streaked onto NGM plates. After 6 days at 37°C, colonies were observed in plates streaked with untreated CBX102 and UV336; however, no colonies were observed on plates streaked with heat-inactivated bacteria. For assays in which lawn avoidance was prevented, bacterial lawns were prepared as described above, but the culture was spread to cover the whole plate.

Unless otherwise stated, adult animals were transferred from *E. coli* OP50 plates to infection plates and F1 progeny were scored for lawn avoidance (as described below) and the Dar phenotype. Experiments were performed in triplicate and repeated at least three times.

Pathogen load and pathogen clearance were assessed by labelling *M. nematophilum* using SYTO13. SYTO13 staining was performed as described previously [22]. Following incubation with SYTO13, animals were either transferred to unseeded plates, for clearance assays as described previously [15], or mounted for imaging and processed as described in Anderson *et al.* [15].

### (c) Lawn avoidance assays

Lawn avoidance assay plates were prepared by seeding standard NGM plates with 200 µl of 10% *M. nematophilum* CBX102 or UV336 diluted in *E. coli* OP50. Control plates were seeded with 200 µl of *E. coli* OP50. Plates were allowed to dry at room temperature overnight. Three adult animals were transferred from *E. coli* OP50 plates to lawn avoidance plates and allowed to lay eggs for between 4 and 8 h. Adult animals were removed and F_1_ progeny were allowed to develop at 20°C. Lawn avoidance (percentage of animals not on the bacterial lawn) was scored when animals reached adulthood. Avoidance index was calculated by dividing the percentage of animals avoiding CBX102-contaminated lawns by the percentage of animals avoiding *E. coli* OP50 lawns. Experiments were performed in triplicate and repeated at least three times.

### (d) Lawn-leaving assays

Lawn-leaving assays were adapted from the male-leaving assay described by Lipton *et al.* [23]. Approximately 20 µl of bacterial culture (OD_600_ = 0.6) was seeded in the centre of 90 mm plates containing 13 ml of NGM. Plates were allowed to dry overnight at room temperature and individual 1-day-old adults were transferred to the centre of the bacterial lawn. Ten plates were prepared for each condition being tested. Plates were kept at 20°C and scored for leaving every hour. Animals were scored as leavers if they, or their tracks, could be seen 1 cm or less from the edge of the plate. Animals scored as leavers were excluded from further analysis. Experiments were repeated at least three times.

### (e) Growth rate assays

Bacterial lawns were prepared as described above. Eggs were obtained by bleaching [24] and allowed to hatch overnight in M9 at 20°C to obtain synchronized populations of L1 animals. Animals were transferred to infection plates as L1s. Approximately 50 L1s were transferred to each plate. After 48 h, the age of animals was determined by categorizing animals into developmental stages (L1–L4 and adult). The age of L4 animals was further categorized based on the development of the vulval as described in MacNeil *et al.* [25]. Experiments were performed in triplicate and repeated at least three times.

### (f) BUS-1 transgenes

pMGN16 (*bus-1p*∷BUS-1cDNA∷GFP) (a gift from Jonathan Hodgkin, University of Oxford) was injected into *bus-1(e2678)* at 10 ng µl^−1^. *impEx58* contains an extrachromosomal version of pMGN16. To express the BUS-1 cDNA specifically in rectal epithelial cells, the BUS-1 cDNA was subcloned into a vector containing a 1.3 Kb fragment of the *egl-5* promoter that drives GFP expression in B, K, F, U, P12.pa and three posterior body wall muscles [26] (pRJM205). pRJM205 was injected into *bus-1(e2678)* at 10 ng µl^−1^ and *impEx70* contains an extrachromosomal version of this plasmid.

### (g) TAX-2 transgenes

Plasmids expressing the TAX-2 cDNA under the control of *flp-6*, *gcy-32*, *gcy-8* or *flp-17* promoters to drive TAX-2 expression in ASE, AQR, PQR and URX, ADF or BAG, respectively, were a gift from Mario de Bono (MRC Laboratory for Molecular Biology). These plasmids were injected into *tax-2(p694)* at 20 ng µl^−1^. *impEx71*, *impEx72* and *impEx73* contain extrachromosomal versions of *flp-6p*∷TAX-2cDNA;*gcy-32p*∷TAX-2cDNA.

### (h) *S. marcescens* infection and survival and lawn avoidance assay

*S. marcescens* infections, survival and lawn avoidance assays were performed as described previously [11]. For survival assays, live animals were transferred to new NGM plates seeded with *S. marcescens* Db10 daily. Experiments were performed in triplicate and repeated at least three times.

### (i) Statistical analysis

In all cases, statistical analysis was performed using Prism 6 (GraphPad Software). Normality was determined using a D'Agostino-Pearson omnibus normality test. Pathogen load was compared using a Mann–Whitney test. Dar data were compared using a Mann–Whitney test or one-way ANOVA followed by Tukey's HSP post hoc multiple comparison test. The avoidance index was calculated by dividing the percentage of animals avoiding CBX102-contaminated lawns by the percentage of animals avoiding *E. coli* OP50 lawns, and data were compared using a one-way ANOVA followed by Tukey's HSP post hoc multiple comparison test (for data with a Gaussian distribution) or the Kruskal–Wallis test followed by Dunn's multiple comparison test (for non-Gaussian data). Lawn avoidance, leaving assay, *S. marcescens* avoidance, growth rate and pathogen clearance data were compared using a two-way ANOVA followed by Tukey's HSP post hoc multiple comparison test (for lawn avoidance, leaving assay, *S. marcescens* avoidance and growth rate) or Sidak's multiple comparison test (for pathogen clearance data). Significance levels are indicated on figures and in the text as follows: *p ≤ 0.05, **p ≤ 0.01, ***p ≤ 0.001, ****p ≤ 0.0001, n.s. *p* > 0.05.

## 3. Results

### (a) *C. elegans* avoids lawns contaminated with pathogenic *M. nematophilum*

**Figure 1.**
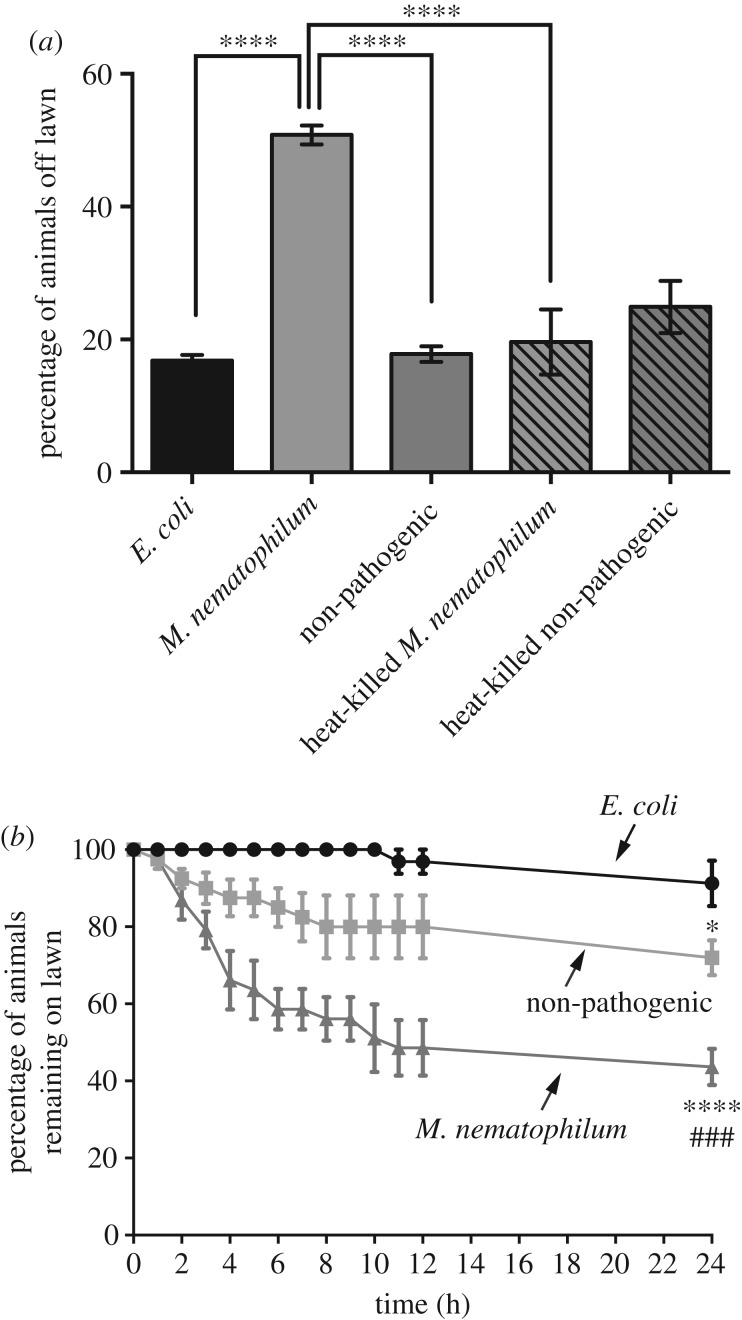
*C. elegans* avoids pathogen-contaminated lawns. (*a*) The percentage of wild-type animals avoiding bacterial lawns. Contamination of bacterial lawns with *M. nematophilum* increases lawn avoidance to 50.8% when compared with control *E. coli* lawns (16.8%). By contrast, lawn avoidance was not significantly increased when bacterial lawns were contaminated with either non-pathogenic or heat-killed *M. nematophilum*. (*b*) Lawn-leaving assays [23] were used to determine whether monoaxenic lawns of *E. coli* OP50, *M. nematophilum* or non-pathogenic *M. nematophilum* alter *C. elegans* behaviour. Wild-type animals remain on lawns of *E. coli* for the duration of the assay. By contrast, 28.1% of wild-type animals leave pure non-pathogenic *M. nematophilum* lawns. The percentage of animals leaving bacterial lawns is further increased to 56.4% when leaving assays are performed using pure *M. nematophilum* lawns. * indicates significance relative to *E. coli*. # indicates significance relative to non-pathogenic *M. nematophilum* (*p ≤ 0.05, ***p ≤ 0.001, ****p ≤ 0.0001. See Material and methods for details of statistical analysis).

We and others have previously observed that wild-type *C. elegans* avoids lawns of its bacterial food *E. coli* OP50 contaminated with *M. nematophilum* [14]. To quantify this effect, we scored lawn avoidance for wild-type animals grown for one generation on contaminated bacterial lawns (figure 1*a*). Approximately 51% of wild-type animals avoided lawns composed of the standard *C. elegans* bacterial food (*E. coli*, OP50) contaminated with 10% *M. nematophilum*. By contrast, only 17% animals avoided uncontaminated *E. coli* bacterial lawns (figure 1*a*). This lawn avoidance behaviour required live bacteria because this increase in lawn avoidance was not observed when *E. coli* lawns were contaminated with heat-killed *M. nematophilum* (figure 1*a*).

Food abundance and food quality have also been shown to regulate lawn occupancy and animals leave bacterial lawns when food levels are low or of poor quality [27,28]. Therefore, the avoidance phenotype that we observed could be due to changes in *C. elegans* behaviour caused by either decreased availability/quality in contaminated bacterial lawns or the pathogenic nature of *M. nematophilum*. To distinguish between these two possibilities, we made use of a non-pathogenic form of *M. nematophilum* [29], which fails to trigger the *C. elegans* immune response [30]. We first compared the behaviour of *C. elegans* exposed to monoaxenic lawns of *E. coli* OP50, *M. nematophilum* or the non-pathogenic form of *M. nematophilum* using a lawn-leaving assay. Individual adult animals were transferred to small bacterial lawns seeded in the centre of 90 mm plates and lawn leaving was assessed over a 24 h period. Animals were scored as having left the lawn if they, or their tracks, could be seen within 1 cm of the edge of the plate. Animals placed on *E. coli* lawns remained feeding on these lawns for the duration of the experiment (figure 1*b*); however, animals exposed to *M. nematophilum* left the lawns with only 43.6% of animals remaining on the lawns after 24 h (figure 1*b*). Using lawns of non-pathogenic *M. nematophilum*, we observed some animals leaving lawns (figure 1*b*) although significantly more animals remained on these non-pathogenic lawns after 24 h than on pathogenic *M. nematophilum* lawns (figure 1*b*, compare 43.6% remaining on *M. nematophilum* lawns at 24 h with 71.9% remaining on non-pathogenic *M. nematophilum* lawns). These data suggest that, although the pathogenic nature of *M. nematophilum* accounts for the majority of the lawn-leaving phenotype, differences in food quality between *E. coli* OP50 and *M. nematophilum* are sufficient to alter *C. elegans* behaviour.

To determine whether the change in behaviour caused by differences in food quality between *M. nematophilum* and *E. coli* had a significant effect on the avoidance of contaminated lawns, we performed lawn avoidance assays using non-pathogenic *M. nematophilum*. We did not observe any significant differences in lawn avoidance between *E. coli* OP50 and lawns contaminated with non-pathogenic *M. nematophilum* (figure 1*a*), indicating that the pathogenicity of *M. nematophilum*, and not differences in food quality or abundance on contaminated lawns, was responsible for the lawn avoidance behaviour that we observed on *M. nematophilum-*contaminated lawns.

### (b) Pathogen avoidance decreases pathogen load and increases pathogen clearance rates

What role does pathogen avoidance play in protecting *C. elegans* from infection by *M. nematophilum*? To address this question, we modified our experimental conditions to prevent pathogen avoidance by spreading contaminated bacterial lawns over the entire agar surface (full plates) and assessing host responses to infection with *M. nematophilum* by scoring the Dar phenotype [31], pathogen load [15], pathogen clearance rate [15] and growth rate [25,31].

**Figure 2.**
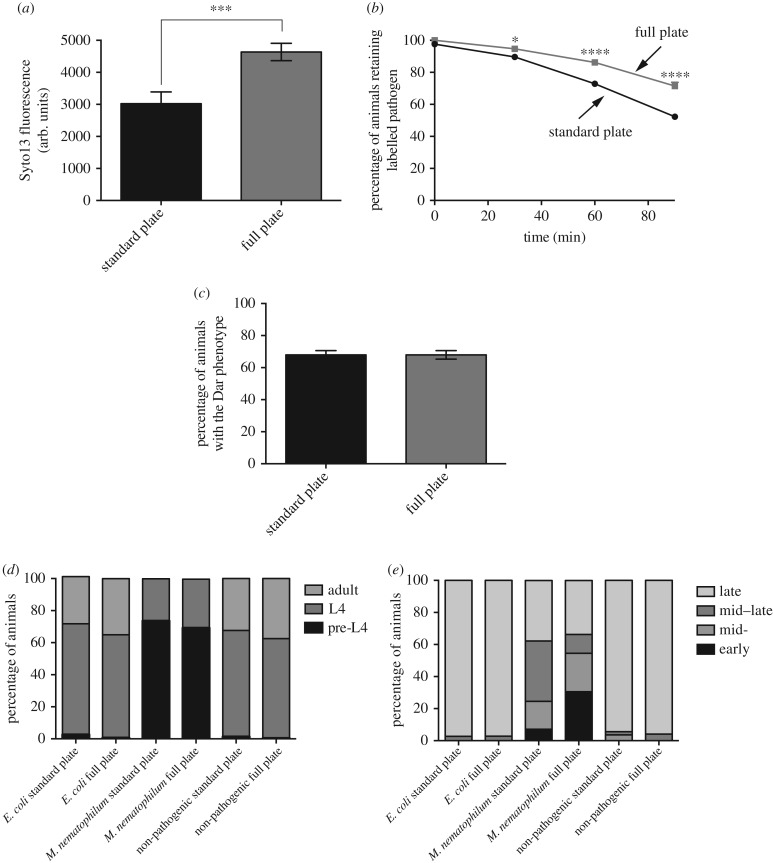
Avoidance of contaminated bacterial lawns protects *C. elegans* from infection by reducing pathogen load and increasing pathogen clearance. (*a*) Pathogen load under avoidance (standard plate) and non-avoidance (full plate) conditions. Pathogen load was assessed by measuring the fluorescence of SYTO13-labelled *M. nematophilum* in the rectal opening of wild-type animals. SYTO13 fluorescence was increased from 3023 arbitrary units on standard plates to 4634 arbitrary units on full plates. (*b*) The clearance of SYTO13-labelled pathogen from the rectal opening of animals infected under avoidance (standard plate) and non-avoidance (full plate) conditions. Labelled pathogen is cleared more quickly under avoidance conditions than non-avoidance conditions, with 52.3% of animals on standard plates and 71.4% of animals on full plates retaining labelled pathogen at 90 min. (*c*) The Dar phenotype of animals grown on lawns contaminated with 0.05% *M. nematophilum* under avoidance (standard plate) and non-avoidance (full plate) conditions. No significant difference was observed. (*d*) Developmental stage of animals grown on contaminated bacterial lawns under avoidance (standard plate) and non-avoidance (full plate) conditions. Animals were transferred to plates as L1s and the developmental stage was assessed 48 h after transfer. Infection with *M. nematophilum* decreases the proportion of animals reaching L4/adult stages; however, the proportion of animals classified as pre-L4 was not significantly altered by manipulating avoidance conditions. (*e*) L4 animals were further classified as early, mid, mid–late or late L4 according to their vulval development [25]. L4 development was delayed in animals grown on *M. nematophilum*-contaminated bacterial lawns and this delay was enhanced when animals were prevented from avoiding contaminated bacterial lawns. * indicates significance relative to standard plates (*p ≤ 0.05, ***p ≤ 0.001, ****p ≤ 0.0001. See Material and methods for details of statistical analysis).

Preventing pathogen avoidance increased pathogen load, as determined by measuring levels of SYTO-13 labelled *M. nematophilum* in the rectal opening (figure 2*a*), and decreased the rate of clearance of this labelled pathogen from the rectal opening (figure 2*b*) when compared with animals infected under standard avoidance conditions (figure 2*a*,*b*). Taken together, these data indicate that animals that were unable to avoid contaminated bacterial lawns were more severely infected than control animals.

When infected with *M. nematophilum*, animals mount a protective response to infection which includes the deformed anal region, or Dar, phenotype. To determine whether the increased pathogen avoidance altered this protective response, we scored the Dar phenotype in animals grown under standard avoidance conditions and full plate conditions. Under our standard infection conditions of 10% *M. nematophilum*, almost all animals (greater than 97%) become Dar on standard plates making it difficult to determine whether inability to avoid contaminated bacteria could alter the Dar phenotype (A. Anderson, data not shown). Therefore, we modified our infection conditions, so that *E. coli* OP50 lawns were contaminated with 0.05% *M. nematophilum* [15]. Under standard avoidance conditions, 68% of animals had the Dar phenotype on these lawns and we observed no difference when animals were infected under full plate conditions (figure 2*c*). Therefore, failure to avoid contaminated bacterial lawn does not have an observable effect on this protective response.

Animals infected with *M. nematophilum* grow more slowly than those grown on *E. coli* OP50 [31]; therefore, we asked whether preventing pathogen avoidance and increasing pathogen load would exacerbate this growth delay. To do this, we scored the developmental stage of animals grown from L1 stage on standard and full plates. After 48 h on standard *E. coli* lawns or lawns contaminated with non-pathogenic *M. nematophilum*, almost all animals had reached L4 or adult stages and this was not significantly altered by preventing lawn avoidance (figure 2*d*). However, after 48 h, only 26% of animals grown on standard lawns contaminated with *M. nematophilum* had reached the L4 stage (figure 2*d*). The percentage of animals reaching the L4 stage was not significantly altered by preventing pathogen avoidance (figure 2*d*). These L4 animals were further classified according to whether they had reached the early, mid- or late L4 stage using vulval development [25]. We found that 38% of animals on standard plates were at the mid–late L4 stage (figure 2*e*) after 48 h on standard *M. nematophilum*-contaminated lawns. By contrast, when animals were unable to avoid *M. nematophilum-*contaminated lawns, the percentage of animals at the mid–late L4 stage was decreased to 12% while the percentage of animals at the early L4 was increased from 7% on standard plates to 31% on full plates (figure 2*e*). Taken together, these results demonstrate that pathogen avoidance protects animals from the detrimental effects of infection on growth by decreasing pathogen load.

### (c) Pathogen avoidance requires signalling in sensory neurons

**Figure 3.**
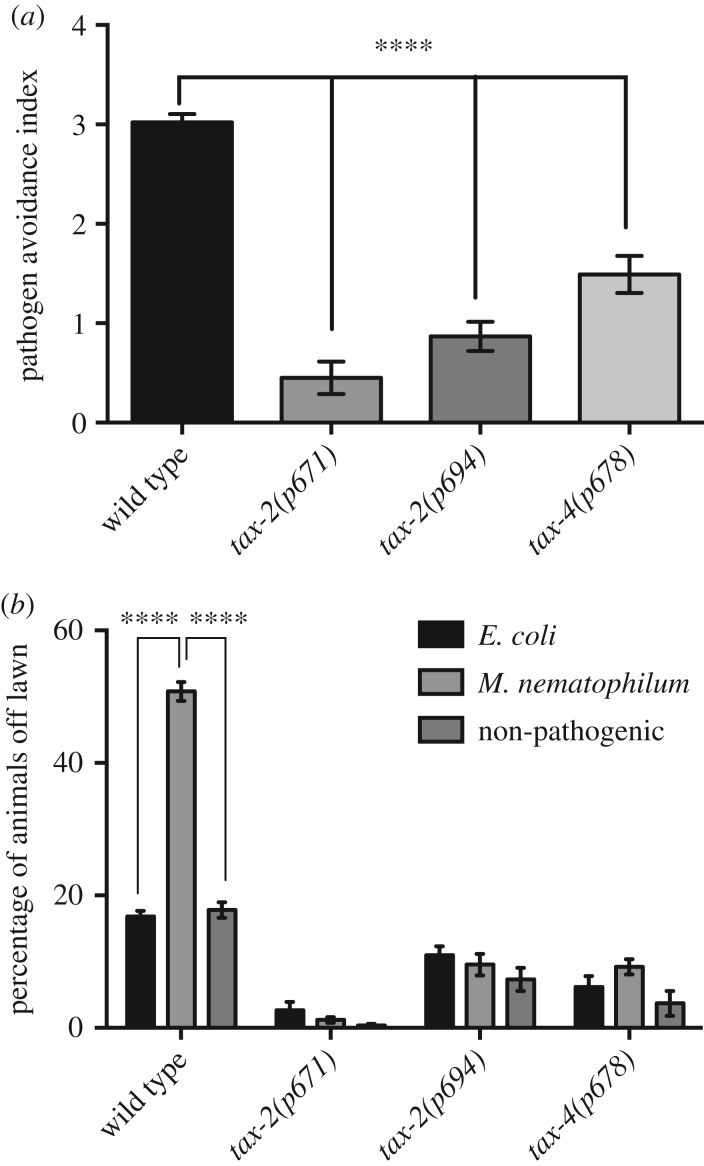
Avoidance of pathogen-contaminated lawns requires TAX-2/4. (*a*) Lawn avoidance index was calculated by dividing the percentage of animals avoiding *M. nematophilum*-contaminated lawns by the percentage of animals avoiding OP50 *E. coli* lawns. Pathogen avoidance index of wild-type, *tax-2(p671)*, *tax-2(p694)* and *tax-4(p678)* animals. (*b*) The percentage of *tax-2(p671)*, *tax-2(p694)* and *tax-4(p678)* animals avoiding *M. nematophilum-*contaminated bacterial lawns is decreased when compared with wild-type controls. (**p ≤ 0.01, ***p ≤ 0.001, ****p ≤ 0.0001. See Material and methods for details of statistical analysis).

Previous studies have shown that the cyclic nucleotide-gated channel encoded by *tax-2* and *tax-4* is required for the avoidance of *S. marcescens* [11]. Furthermore, alleles of *tax-2* were isolated in a screen for genes required for the host response to *M. nematophilum* infection and shown to be required for choice between *E. coli OP50* and *M. nematophilum* [14]. To determine whether the TAX-2/TAX-4 channel was required for the avoidance of *M. nematophilum*-contaminated bacterial lawns, we performed lawn avoidance assays using *tax-2* and *tax-4* mutants and calculated a pathogen avoidance index for each strain (see Material and methods for details), which allowed us to compare the ability of different strains to initiate avoidance behaviours in response to the presence of pathogenic bacteria. When compared with wild-type animals, we observed no difference in the ability of *tax-2(p671)* and *tax-4(p678)* animals to avoid *E. coli* lawns or lawns contaminated with non-pathogenic *M. nematophilum* (figure 3*b*). However, we observed a significant decrease in both the percentage of *tax-2(p671)* and *tax-4(p678)* animals avoiding *M. nematophilum*-contaminated lawns (figure 3*b*) and the pathogen avoidance index of *tax-2(p671)* and *tax-4(p678)* animals (figure 3*a*), suggesting that neurons expressing TAX-2 and TAX-4 are required for the avoidance of *M. nematophilum*.

**Figure 4.**
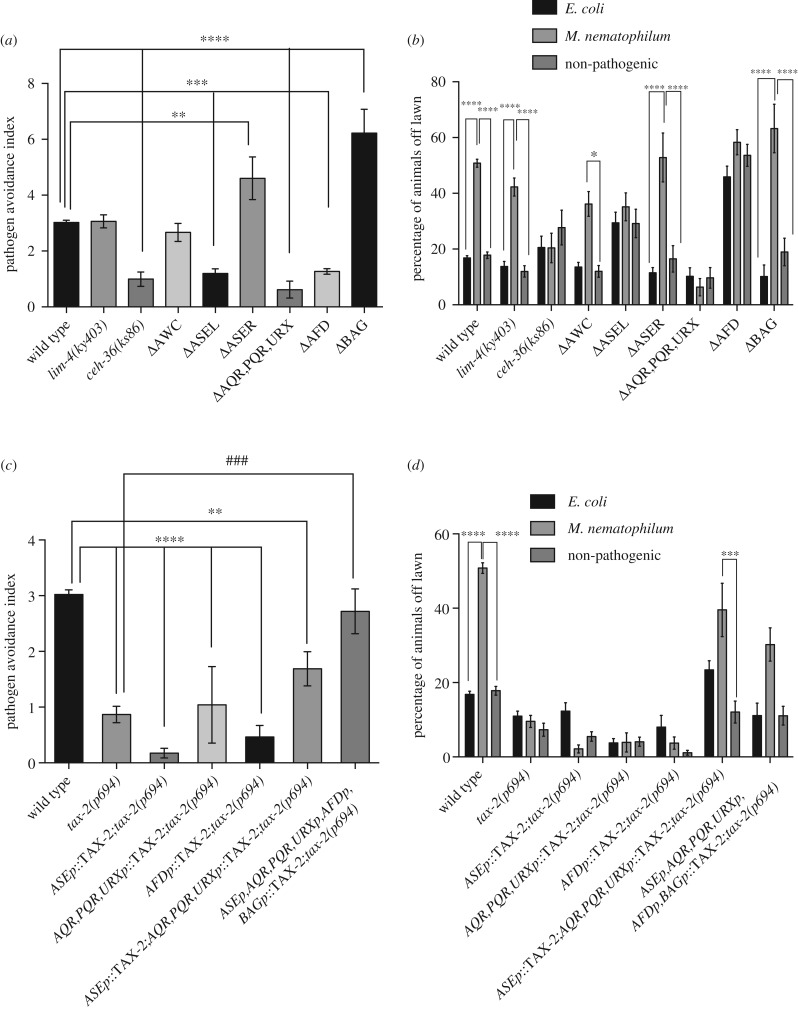
Avoidance of pathogen-contaminated lawns requires signalling in multiple sensory neurons. (*a*) Pathogen avoidance index of animals lacking functional sensory neurons. The avoidance index was decreased when compared with wild type in animals lacking ASEL and AWC (*ceh-36*), ASEL alone (ΔASEL), AQR, PQR and URX (ΔAQR, PQR, URX) or AFD alone (ΔAFD). Pathogen avoidance index was increased when compared with wild type in animals lacking ASER alone (ΔASER) or BAG (ΔBAG). (*b*) Percentage of animals lacking functional sensory neurons avoiding *M. nematophilum-*contaminated bacterial lawns. The percentage of animals avoiding *M. nematophilum*-contaminated lawns was decreased when compared with wild type in animals lacking ASEL and AWC (*ceh-36*), ASEL alone (ΔASEL) or AQR, PQR and URX (ΔAQR, PQR and URX). The percentage of animals avoiding *E. coli* lawns was increased in animals lacking AFD (ΔAFD). (*c*) Pathogen avoidance index of *tax-2(p694)* cell-specific rescuing lines. The decreased pathogen avoidance index of *tax-2(p694)* was rescued by expression of TAX-2 cDNA in ASE, AQR, PQR, URX, AFD and BAG and partly by expression in ASE, AQR, PQR and URX but not by expression of TAX-2 cDNA in ASE alone, AFD alone or AQR, PQR and URX. (*d*) Percentage of *tax-2(p694)* cell-specific rescuing lines avoiding *M. nematophilum*-contaminated bacterial lawns. The lawn avoidance defect of *tax-2(p694)* was rescued by expression of TAX-2 cDNA in ASE, AQR, PQR, URX, AFD and BAG or by expression in ASE, AQR, PQR and URX but not by expression of TAX-2 cDNA in ASE alone, AFD alone or AQR, PQR and URX. See electronic supplementary material, table S1, for details of strains used. * indicates significance relative to wild type. # indicates significance relative to *tax-2(p694)* (**p ≤ 0.01, ***p ≤ 0.001, ****p ≤ 0.0001. See Material and methods for details of statistical analysis).

The TAX-2/TAX-4 channel is essential for the function of at least nine pairs of amphid chemosensory neurons [32,33]. One of these, AWB, is required to stimulate the avoidance of *S. marcescens* [11]; however, two lines of evidence suggest that AWB is not required for the avoidance of *M. nematophilum*-contaminated lawns. Firstly, *lim-4(ky403)* animals, which lack functional AWB neurons, have a wild-type avoidance response to bacterial lawns contaminated with *M. nematophilum* (figure 4*a*,*b*). Secondly, an additional *tax-2* allele, *tax-2(p694)*, resulted in a pathogen avoidance defect similar to that observed in *tax-2(p671)* animals (figure 3*a*,*b*). *tax-2(p694)* mutants carry a deletion in *tax-2* that has been demonstrated to affect TAX-2 function only in AQR, PQR, URX, AFD, ASE and BAG chemosensory neurons [32]. The fact that *tax-2(p694)* animals are defective in avoidance of *M. nematophilum*-contaminated lawns implicates one or more of these neurons in this pathogen avoidance response.

Which of these neurons is required for the avoidance of *M. nematophilum* contamination? To address this question, we assessed the consequences of functionally ablating AFD alone, ASE alone, BAG alone or AQR, PQR and URX on avoidance of *M. nematophilum*-contaminated lawns. Cell-specific expression of the *egl-1* cell death gene to ablate BAG neurons or ablation of ASER using cell-specific expression of caspase3 resulted in an increase in the pathogen avoidance index of these strains when compared with wild-type controls (figure 4*a*); however, this effect may be due, at least in part, to decreased avoidance of *E. coli* lawns, or increased variability in the avoidance response in these strains because we observed that the avoidance of *M. nematophilum-*contaminated bacterial lawns by these strains was not significantly different from wild type (figure 4*b*). Conversely, genetically disrupting ASEL (using *ceh-36(ks86)* mutants), or cell-specific expression of caspase3 to ablate ASEL alone, AFD alone or AQR, PQR and URX, decreased the pathogen avoidance index (figure 4*a*). *ceh-36(ks86)* are defective in the development of both ASEL and AWC; therefore we used animals expressing caspase3 in AWC to confirm that ablation of AWC did not affect lawn avoidance (figure 4*a*,*b*). Since the ability of AQR-, PQR- and URX-ablated animals to avoid *E. coli* lawns is indistinguishable from wild type and the percentage of animals avoiding *M. nematophilum*-contaminated lawns is significantly decreased when compared with wild type (figure 4*b*), the decreased pathogen avoidance index we observed in these animals is likely to reflect a specific defect in pathogen avoidance caused by the loss of AQR, PQR or URX. However, the decreased pathogen avoidance index in AFD-ablated animals appears to be caused by a basal avoidance defect because we failed to observed a significant difference between the percentage of animals avoiding *M. nematophilum*-contaminated lawns in wild-type and AFD-ablated animals and instead observed a significant increase in the percentage of animals avoiding *E. coli* lawns in this strain when compared with wild-type animals (figure 4*b*). Ablation of ASEL also resulted in a slight defect in basal avoidance because there was a small but significant increase in the percentage of ASEL-ablated animals avoiding *E. coli* lawns when compared with wild-type controls (figure 4*b*). However, ASEL also appears to be defective in pathogen avoidance because the percentage of animals avoiding *M. nematophilum*­-contaminated lawns was decreased when compared with wild-type controls (figure 4*b*). Taken together, these results suggest that ASEL and/or AQR, PQR and URX are required for the avoidance of pathogen-contaminated lawns while AFD is required to regulate basal lawn avoidance.

A number of sensory neurons required for pathogen avoidance have been identified [6,11,19,34]. In some cases, these neurons appear to regulate the avoidance of more than one pathogen; for example AWB is required for the avoidance of both *S. marcescens* and *P. aeruginosa* [6,11]. To determine whether ablation of ASEL alone, AFD alone or AQR, PQR and URX altered *C. elegans* ability to avoid other pathogens, we performed lawn avoidance assays using *S. marcescens* Db10. Although some avoidance of *E. coli* OP50 was observed in animals lacking AFD alone (electronic supplementary material, figure S1), confirming our previous observations (figure 4*b*) that AFD regulates basal avoidance, ablation of these neurons individually did not have any effect on the ability of these animals to avoid *S. marcescens* (electronic supplementary material, figure S1). These experiments confirm that animals lacking ASEL alone or AQR, PQR and URX retain the ability to initiate avoidance behaviours in the presence of some noxious stimuli and demonstrate that these neurons are not required for the avoidance of all pathogens, raising the possibility that these neurons may be sensing pathogen-specific cues to initiate avoidance behaviours.

To begin to understand how signalling in these neurons regulates the avoidance of contaminated bacterial lawns, we sought to rescue the pathogen avoidance defect of *tax-2(p694)* mutants by expressing the TAX-2 cDNA from cell-specific promoters. Expression of TAX-2 in ASE, AFD, BAG, AQR, PQR and URX was sufficient to rescue *tax-2(p694)* (figure 4*c*,*d*); however, expression of TAX-2 in ASE alone, AFD alone or AQR, PQR and URX was unable to restore pathogen avoidance in *tax-2(p694)* animals (figure 4*c*,*d*), indicating that TAX-2 is required in multiple neurons to promote pathogen avoidance. To begin to address the combinations of neurons in which TAX-2 is required, we attempted to rescue *tax-2(p694)* mutants by expressing the TAX-2 cDNA in ASE, AQR, PQR and URX. Expression of TAX-2 in ASE, AQR, PQR and URX increased the pathogen avoidance index of *tax-2(p694)* animals (figure 4*c*) and restored the percentage of *tax-2(p694)* animals avoiding *M. nematophilum*-contaminated lawns to levels that were indistinguishable from wild type (figure 4*d*), indicating that expression of TAX-2 in these neurons was sufficient to rescue *tax-2(p694).* However, the pathogen avoidance index of these rescued animals was significantly lower than in wild-type animals (figure 4*c*). This difference most likely reflects an increase in the ability of these animals to avoid lawns of *E. coli* (figure 4*d*).

Taken together, these data indicate that ASEL, AQR, PQR and URX sensory neurons can suppress pathogen avoidance behaviour. Furthermore, signalling in more than one of these neurons is necessary for wild-type avoidance of *M. nematophilum*-contaminated lawns (figure 4), but not avoidance of *S. marcescens* (electronic supplementary material, figure S1).

### (d) Pathogen attachment to epithelial cells is also required for efficient pathogen clearance

Several *tax* mutants are variably Bus (bacterial unswollen) following infection with *M. nematophilum*, implicating the TAX-2/TAX-4 channel in regulation of the Dar phenotype [14]. The Bus phenotype can be caused by failure to *M. nematophilum* to adhere to the rectal opening and has been observed in animals with a defective cuticle including *srf-2*, *srf-3* and *srf-5* [31]. Cuticle defects were observed in *tax-2* and *tax-4* mutants, suggesting that the Bus phenotype may be a consequence of impaired bacterial adherence in these animals [31]. Does impaired bacterial adherence also contribute to the pathogen avoidance defect observed in *tax-2* and *tax-4* mutants?

**Figure 5.**
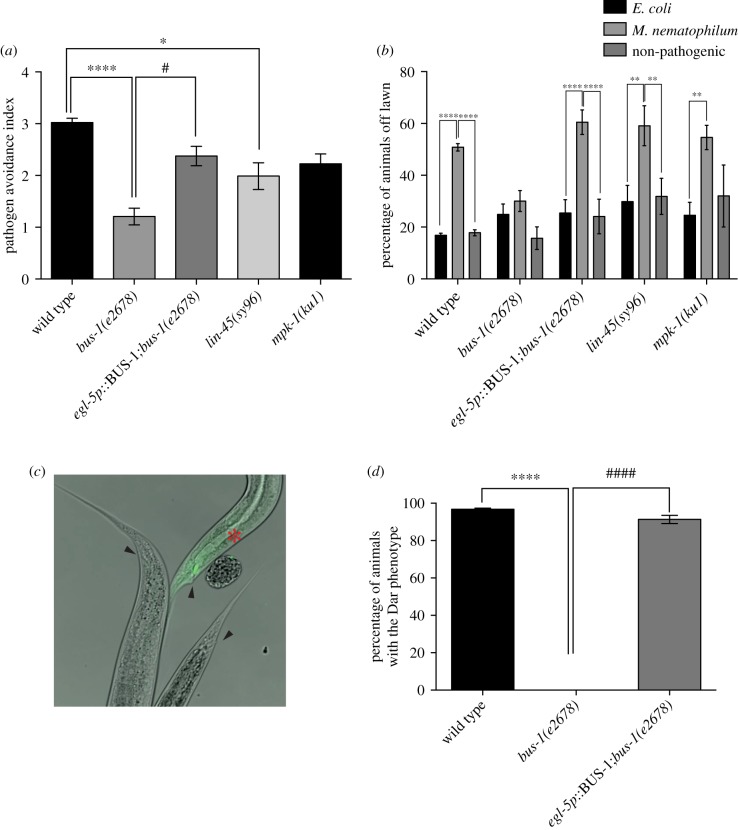
Efficient avoidance of pathogen-contaminated lawns requires pathogen attachment to rectal epithelial cells. (*a*) Pathogen avoidance index is decreased in *bus-1(e2678)* and *lin-45(sy96)* animals, but not in *mpk-1(ku1)* animals. (*b*) The percentage of *bus-1(e2678)* animals avoiding *M. nematophilum-*contaminated bacterial lawns is significantly decreased when compared with wild type. The *bus-1(e2678)* avoidance defect can be rescued by expression of BUS-1 cDNA in rectal epithelial cells (*egl-5p*∷BUS-1;*bus-1(e2678)*) (*a* and *b*). (*c*,*d*) Expression of BUS-1 cDNA in the rectal epithelial cells of *bus-1(e2678)* using a fragment of the *egl-5* promoter (*egl-5p*∷BUS-1;*bus-1(e2678)*) rescues the Bus phenotype of *bus-1(e2678)* animals. (*c*) SYTO13-labelled *M. nematophilum* can be observed attaching to the rectal opening of transgenic (labelled with red asterisk), but not *bus-1(e2678)* animals. Rectal openings are indicated with black arrowheads. * indicates significance relative to wild type. # indicates significance relative to *bus-1(e2678)* (*p ≤ 0.05, ****p ≤ 0.0001. See Material and methods for details of statistical analysis).

To test the hypothesis that impaired bacterial adherence could cause pathogen avoidance defects, we sought to determine the lawn avoidance phenotype of a *C. elegans* Bus mutant defective in *M. nematophilum* adherence. *bus-1* encodes an integral membrane *O*-acyltransferase which is expressed in *C. elegans* neurons and rectal epithelial cells and required for *M. nematophilum* adherence to the rectal opening [35]. *M. nematophilum* cannot be observed adhering to the rectal opening of *bus-1(e2678)* mutants, and these animals fail to become Dar following infection [35]. *bus-1(e2678)* animals failed to avoid bacterial lawns contaminated with *M. nematophilum* (figure 5*a*,*b*). By contrast, we observed a wild-type avoidance response to *S. marcescens* in *bus-1(e2678)* animals (electronic supplementary material, figure S2A), suggesting that the role for *bus-1* in avoidance is pathogen-specific and that *bus-1* does not have a general role in avoiding noxious stimuli. This is consistent with the observation that survival of *bus-1(e2678)* animals infected with *S. marcescens* was not significantly different from wild-type survival (electronic supplementary material, figure S2B).

BUS-1 is expressed in neurons in the head and tail and in rectal epithelial cells [35]. Although cell-specific rescue of *bus-1(e2678)* has not been reported, the observations that the *C. briggsae bus-1* gene is also expressed in the vulva and *M. nematophilum* can be observed adhering to the vulva of *C. briggssae* but not *C. elegans* [35] suggest that BUS-1 expression in the rectal epithelium is required for bacterial adherence. To determine where BUS-1 expression in the rectal epithelium was required to promote pathogen avoidance, we attempted to rescue the pathogen avoidance defect of *bus-1(e2678)* by expressing the BUS-1 cDNA under the control of a fragment of the *egl-5* promoter that drives expression in K, F, U, B and P12.pa rectal epithelial cells as well as three posterior body wall muscles [26]. *bus-1(e2678)* animals expressing the BUS-1cDNA from the *egl-5* promoter were Dar, and SYTO13-labelled *M. nematophilum* could be observed adhering to the rectal opening (figure 5*c*,*d*), confirming that expression in the rectal epithelium was sufficient to rescue bacterial adherence in *bus-1(e2678)*. This rescuing transgene was also sufficient to rescue the *bus-1(e2678)* lawn avoidance defect (figure 5*a*,*b*), indicating that impaired bacterial adherence in these animals contributes to pathogen avoidance.

The Dar phenotype requires ERK/MAPK signalling in the rectal epithelium [21,22]. This signalling pathway acts downstream of bacterial adherence because *lin-45(sy96)* and *mpk-1(ku1)* animals lacking components of the ERK/MAPK pathway fail to become Dar despite *M. nematophilum* attaching to the rectal opening [22]. To determine whether bacterial adherence, the Dar phenotype or both was required for wild-type levels of pathogen avoidance, we performed lawn avoidance assays using *lin-45(sy96)* and *mpk-1(ku1)*. Both *lin-45(sy96)* and *mpk-1(ku1)* animals grow poorly on *M. nematophilum*, making it difficult to score pathogen avoidance in these strains; however, we observed a small decrease in the pathogen avoidance index in the *lin-45(sy96)* animals when compared with wild type (figure 5*a*). The pathogen avoidance index decrease observed in *lin-45(sy96)* animals was significantly weaker than that observed in the *bus-1(e2678)* animals and is most likely a consequence of increased basal avoidance in this strain (figure 5*b*), suggesting that although the Dar phenotype may play a small role in regulating pathogen avoidance, bacterial adherence is required for wild-type levels of pathogen avoidance.

## 4. Discussion

### (a) Avoidance of contaminated food protects *C. elegans* from infection by reducing pathogen load

Understanding the importance of pathogen avoidance in protecting animals from infection in their natural environment is challenging and, consequently, much evidence for the role of pathogen avoidance in disease prevention is anecdotal. To assess the contribution of pathogen avoidance to disease prevention, controlled experimental models in which avoidance behaviour can be manipulated and the effect on infection outcome assessed are required. *C. elegans* is ideally suited to such investigation because avoidance can be prevented easily by spreading pathogenic bacteria, or bacteria contaminated with pathogens, over the entire experimental arena [9]. The effect of these manipulations can easily be assessed by measuring changes in pathogen load using labelled pathogens [22,36], expression of infection-regulated genes [30] or infection outcomes such as death [9] or delayed growth rate [31].

Here, we have established a model of contaminated food avoidance using *C. elegans* propagated on *M. nematophilum*-contaminated bacterial lawns. We find that, in agreement with previous studies [14], *C. elegans* avoids lawns contaminated with pathogenic *M. nematophilum*. Using a non-pathogenic form of *M. nematophilum*, we show that, although *C. elegans* is less attracted to *M. nematophilum* than *E. coli* OP50, avoidance of contaminated bacterial lawns is mediated by pathogenic elements of *M. nematophilum* and not by decreased food availability or poor food quality.

*M. nematophilum* establishes a persistent infection in *C. elegans*, which results in slowed growth [31]. We find that avoidance of *M. nematophilum-*contaminated bacterial lawns protects *C. elegans* from the deleterious effects of *M. nematophilum* infection because we observed that, under conditions where wild-type animals were unable to avoid pathogen-contaminated lawns, their growth was further slowed. This is consistent with previous results obtained using monoaxenic lawns of pathogenic *P. aeruginosa* where manipulating experimental conditions to prevent avoidance of *P. aeruginosa* increases *C. elegans* susceptibility to infection [9].

*M. nematophilum* specifically attaches to the cuticle around the rectal opening of *C. elegans* [22,31] and activates signalling in the rectal epithelial cells as part of a protective cellular immune response that includes changes in morphology of the rectal opening known as the Dar phenotype [31]. Our data suggest that avoidance protects animals by reducing pathogen load independently of the cellular immune response because animals that are unable to avoid pathogen-contaminated lawns retain more labelled pathogen, but do not observably alter their Dar phenotype. Previous studies have also demonstrated a separation of immune signalling and avoidance responses following cellular damage [37]. Further investigation to determine the role of other aspects of the cellular immune response, such as the upregulation of antimicrobial peptide expression, in the avoidance of *M. nematophilum-*contaminated lawns will be required in order to fully exclude a role for cellular immune responses in the regulation of this pathogen avoidance behaviour.

Our results establish *C. elegans* as a model that can be used to genetically dissect the molecular and cellular basis of behaviours that lead to the avoidance of contaminated food and to examine the impact of these behaviours on infection.

### (b) Regulation of multiple sensory neurons is required for the avoidance of contaminated food

The cyclic nucleotide-gated channel encoded by *tax-2* and *tax-4* is essential for the function of many sensory neurons [32,33] and is required for the avoidance of *S. marcescens* [11] and *M. nematophilum* [14]. Here, we show that animals lacking *tax-2* or *tax-4* are defective in their ability to avoid *M. nematophilum­*-contaminated bacterial lawns, implicating sensory neuron signalling in the avoidance of contaminated food. We find that ASEL, AQR, PQR and URX sensory neurons act to promote the avoidance of contaminated bacterial lawns. This is in contrast to previous work by Yook & Hodgkin [14] who demonstrated that *tax-2(p694)* responded in a similar manner to wild-type animals in a bacterial choice assay. Since *tax-2(p694)* has been demonstrated to only affect TAX-2 function in a subset of TAX-2-expressing neurons, their data suggest that the TAX-2/4 channel is required in AWB, AWC, ASJ, ASI, ASG or ASK to mediate the choice between *M. nematophilum* and *E. coli* OP50 [14]. Further work is required to resolve this discrepancy which likely reflects the use of monoaxenic bacterial lawns [14] versus mixed bacterial lawns (this study).

Our results identify a novel role for ASEL in regulating pathogen avoidance; however, URX has previously been shown to regulate aversive olfactory learning of *P. aeruginosa* [38]*.* Interestingly, the neurons that we have identified are also required for the avoidance of other unfavourable environmental conditions [27,39]. Under conditions where food is depleted, ASE, AQR, PQR and URX promote food-leaving behaviour via TAX-2/4, while AFD suppresses the response [27]. Additional neurons, including ASI and ADF, are also required for this response [27], and the role of these neurons in the avoidance of contaminated bacterial lawns remains to be determined.

Although ablation of individual neurons is sufficient to supress pathogen avoidance, our rescue data suggest that efficient pathogen avoidance requires the integration of sensory inputs from multiple neurons because expression of the TAX-2 cDNA in multiple neurons is required to rescue *tax-2(p694)*. The requirement for multiple sensory neurons suggests that *C. elegans* is detecting multiple cues in order to avoid contaminated bacterial lawns. This may include attractive chemosensory cues provided by the *E. coli* to promote lawn retention and feeding as well as repulsive cues from the pathogenic *M. nematophilum*. These microbial cues remain to be identified however, based on the requirement for TAX-2/4 in this response, it is likely that at least some of these cues will involve G-protein signalling. Aerotaxis has been implicated in the regulation of pathogen avoidance with chemosensation of *P. aeruginosa* secondary metabolites by the sensory neuron ASJ altering *C. elegans* aerotaxis behaviour to promote pathogen avoidance [19]. Further experiments are required to elucidate the role of aerotaxis in our model; however, our current study raises the possibility that aerotaxis has a role in regulating the avoidance of *M. nematophilum-*contaminated bacterial lawns because AQR, PQR and URX are activated by high ambient O_2_ [40,41] to promote adaptive food-leaving behaviour [27] while AFD, ASE and BAG act as a CO_2_ sensors to mediate CO_2_ avoidance [39].

### (c) Avoidance of contaminated food requires neuronal and non-neuronal signals

Pathogen attachment to the rectal opening requires expression of the integral O-membrane-linked acyltransferase BUS-1 in rectal epithelial cells. In *bus-1(e2678)* animals, *M. nematophilum* fails to attach and, as a consequence, these animals do not develop the Dar phenotype [35]. Here, we show that BUS-1 is required in the rectal epithelium for wild-type levels of avoidance, revealing a novel role for the rectal epithelium in regulating behaviour and indicating that pathogen attachment is required for both behavioural and cellular responses to infection.

How does bacterial adherence to non-neuronal cells alter behaviour? Although a link between bacterial adherence and regulation of neuronal activity has not previously been demonstrated, the kinetics of aversive learning in avoidance of *P. aeruginosa* suggests that ingestion of the bacteria and subsequent cellular damage are required for lawn avoidance [7]. Furthermore, there is evidence that cellular damage to non-neuronal cells, such as that caused by ingestion of pathogenic bacteria, can modify behaviour via neuroendocrine signalling pathways [37]. Using non-pathogenic bacteria expressing RNAi to knockdown essential genes Melo & Ruvkun [37] demonstrated that disrupting core cellular activities in non-neuronal cells could stimulate avoidance behaviours. This lawn avoidance behaviour requires signalling by the stress-activated JNK–MAPK pathway but not signalling via immune response pathways that are activated by infection with *P. aeruginosa* [37]*.* Adherence of *M. nematophilum* to the rectal opening of *C. elegans* has not yet been reported to cause cellular damage or inactivate essential cellular pathways in the manner described by Melo & Ruvkun [37], and the JNK–MAPK pathway has not been implicated in the host response to *M. nematophilum* infection. However, consistent with the previous observation that immune response pathways are not required for avoidance [37], the ERK–MAPK pathway, which is required for activation of the cellular immune response to *M. nematophilum*, does not appear to be required for avoidance of *M. nematophilum-*contaminated lawns. Further work to elucidate the cellular response to *M. nematophilum* adherence will be required in order to understand the neuroendocrine signals that link bacterial adherence with changes in behaviour.

Our study also raises the possibility that impaired bacterial adherence contributes to the pathogen avoidance phenotype observed in *tax-2* and *tax-4* mutants. *tax-2(p694)* animals have a wild-type Dar response [14]; however, we have shown that these animals are defective in pathogen avoidance, suggesting that impaired bacterial adherence does not contribute to the observed pathogen avoidance defect in this mutant. However, bacterial adherence is impaired in *tax-2(p671)* and *tax-4(p678)* animals [14], and the contribution of impaired bacterial adherence to the pathogen avoidance defect in these animals remains to be established.

## 5. Conclusion

One way in which animals minimize the risk of infection is to reduce their contact with contaminated food. To do this, they must detect the presence of pathogen contamination and integrate this information with attractive food cues and information about their internal nutritional status to elicit an appropriate behavioural response. Here, we have established a model of contaminated food avoidance using the invertebrate model *C. elegans*. The genetic tractability of this model provides a unique opportunity to dissect these behaviours at molecular and cellular levels in order to gain basic insights into how animals avoid contaminated food. Given the evolutionary importance of avoiding contaminated food, we anticipate that at least some of these mechanisms will be conserved.

## Supplementary Material

Supporting Data

## Supplementary Material

Supplemental figure 1

## Supplementary Material

Supplemental figure 2

## Supplementary Material

Supplemental Table 1

## Data accessibility

The datasets supporting this article have been uploaded as part of the electronic supplementary material.
