# Supplementary material for: Neuronal and non-neuronal signals regulate Caernorhabditis elegans avoidance of contaminated food
Source: Philos Trans R Soc Lond B Biol Sci. 2018 Jun 4;373(1751):20170255. doi: 10.1098/rstb.2017.0255 (PMC6000145; doi:10.1098/rstb.2017.0255)
Supplement: Supplemental Table 1 [file rstb20170255supp4.docx]

**TABLE S1 *C. elegans* strains used in this study**

| Strain | Strain description | Genotype | Source | Reference |
| --- | --- | --- | --- | --- |
| N2 Bristol | Wild type | Wild type | CGC | Brenner 1974 **[21]** |
| PR671 | Lacking TAX-2 function in all TAX-2 expressing neurons | *tax-2(p671)* | CGC | Coburn and Bargmann 1996 **[33]** |
| PR694 | Lacking TAX-2 function in AQR, PQR, URX, AFD, ASE and BAG | *tax-2(p694)* | CGC | Coburn and Bargmann 1996 **[33]** |
| PR678 | Lacking TAX-4 function in all TAX-4 expressing neurons | *tax-4(p678)* | CGC | Komatsu 1996 **[34]** |
| AX2161 | TAX-2 expressed in ASE (ASEp∷TAX-2) | *tax-2(pr694);lin-15(n765);*dbEx724[*flp-6p*∷TAX-2∷SL2∷GFP;*lin-15*(+)] | CGC | Bretscher et. al. 2011 **[41]** |
| AX2159 | TAX-2 expressed in AQR, PQR and URX | [*tax-2*](http://www.wormbase.org/species/c_elegans/gene/WBGene00006525)*(*[*p694*](http://www.wormbase.org/search/variation/p694)*)*; *lin-15(n765);* dbEx723 [[*gcy-32p*](http://www.wormbase.org/search/gene/gcy-32p)::[TAX-2](http://www.wormbase.org/search/gene/tax-2)::SL2::GFP;*lin-15(+)*] | CGC | Bretscher et. al. 2011 **[41]** |
| AX2178 | TAX-2 expressed in AFD | *tax-2(pr694);lin-15(n765);*dbEx726[*gcy-8p*∷TAX-2∷SL2∷GFP;*lin-15*(+)] | CGC | Bretscher et. al. 2011 **[41]** |
| AX2157 | TAX-2 expressed in BAG | *tax-2(pr694);lin-15(n765)*;dbEx722[*flp-17p*∷TAX-2∷SL2∷GFP;*lin-15*(+)] | CGC | Bretscher et. al. 2011 **[41]** |
| AX2164 | TAX-2 expressed in AQR, PQR, URX, AFD, ASE and BAG | *tax-2(pr694);lin-15(n765)*;dbEx725[*flp-6p*∷TAX-2*, flp-17p*∷TAX-2, *gcy-8p*∷TAX-2*, gcy-32p*∷TAX-2;*lin-15*(+)] | CGC | Bretscher et. al. 2011 **[41]** |
| CX3937 | Lacking functional AWB | *lim-4(ky403)* | CGC | Sagasti et. al. 1999 **[44]** |
| FK311 | Lacking functional AWC and ASE | *ceh-36(ks86)* | CGC | Koga and Ohshima 2004 **[45]** |
| PY7502 | Δ AWC | oyIs85 [*ceh-36p*∷CZcaspase3;*ceh-36p*∷caspase3-NZ;*srtx-1p*∷GFP;*unc-122p*∷DsRed] | CGC | Beverly et. al. 2011 **[46]** |
| OH9019 | Δ ASEL | otIs4 [*gcy-7p*∷GFP];otIs253[*ceh-36p*::CZ-caspase3 + *gcy-7p*::caspase3-NZ] | CGC | Ortiz et. al. 2009 **[47]** |
| OH8593 | Δ ASER | ntIs1 [*gcy-5p*∷GFP];otEx3830[*ceh-36p*::CZ-caspase3 + *gcy-5p*::caspase3-NZ] | CGC | Ortiz et. al. 2009 **[47]** |
| CX7102 | Δ AQR, PQR, URX | *lin-15(n765);*qaIs2241 [*gcy-36p*∷egl-1*;gcy-35p*∷GFP*;lin-15*(+)] | CGC | Chang et. al. 2006 **[48]** |
| PR767 | Lacks functional AFD (Δ AFD) | *ttx-1(p767)* | CGC | Mori and Ohshima 1995 **[49]** |
| AX2051 | Δ BAG | dbEx[*gcy-33p∷*egl-1*;unc-122p*∷DsRed] | Mario de Bono (MRC Laboratory for Molecular Biology, Cambridge) | Milward et. al. 2011 **[28]** |
| QT1035 | bus-1 loss-of-function allele | *bus-1(e2678)* | Jonathan Hodgkin (University of Oxford) | Gravato-Nobre et. al. 2008 **[36]** |
| PS427 | lin-45 reduction-of-function allele | *lin-45(sy96)* | CGC | Han 1993 **[50]** |
| QT977 | mpk-1 reduction-of-function allele | *mpk-1(ku1)* | CGC | Lackner and Kim 1998 **[51]** |
